# Successful obstetrical management of 110-day intertwin delivery interval without cerclage: counseling and conservative management approach to extreme asynchronous twin birth

**DOI:** 10.1186/1471-2393-4-23

**Published:** 2004-12-06

**Authors:** Labib M Ghulmiyyah, Salim A Wehbe, Seth A Schwartz, Eric Scott Sills

**Affiliations:** 1 Department of Obstetrics and Gynecology, Atlanta Medical Center; Atlanta GA 30321 USA; 2 Georgia Reproductive Specialists LLC/Reproductive Endocrinology Division, Department of Obstetrics and Gynecology, Atlanta Medical Center, Atlanta GA 30321 USA

## Abstract

**Background:**

This report describes a patient counseling approach and non-surgical management of a dichorionic-diamniotic twin pregnancy where delivery of the second twin followed the delivery of the first by 110 days.

**Case presentation:**

An early transvaginal sonogram at 19 1/2 weeks suggested cervical dilation with protruding amniotic membranes. Tocolytic and antibiotic therapy was initiated; no cerclage was placed. Spontaneous rupture of membranes and cord prolapse occurred 48 h later, resulting in delivery of a stillborn female infant. Conservative management was offered after counseling for possible risks associated with maternal sepsis, need for extended hospitalization, potential for hysterectomy and death. The cervix appeared closed after delivery and the umbilical cord was ligated, with subsequent spontaneous cord retraction *in utero*. Reassuring fetal status was observed for twin B without evidence of contractions or chorioamnionitis. A viable male infant (2894 g) was delivered vaginally at 35 1/2 weeks.

**Conclusions:**

This report outlines a counseling approach useful for patients with premature delivery of one twin, and presents application of conservative obstetrical management principles for the aftercoming twin even when delivery interval is extreme.

## Background

The incidence of twin gestation has increased in recent years, a trend influenced in part by greater utilization of the assisted reproductive technologies. In this report, we describe a conservative approach culminating in vaginal delivery of a viable second twin 110 days after delivery of a preterm stillbirth.

## Case presentation

A 30 year-old non-smoking Caucasian G_3_P_1011 _presented for initial prenatal assessment at six weeks gestation. The conception was established without medical assistance. The patient had no significant medical or surgical history. She underwent an uncomplicated curettage for missed abortion four years before presentation and an uneventful term vaginal delivery occurred two years later.

Transvaginal ultrasound at seven weeks gestation revealed a dichorionic-diamniotic twin pregnancy. At 18 weeks gestation no growth discordance was noted, but cervical length was two cm with funneling. Based on these findings, the patient was counseled about maternal and neonatal risks associated with twin pregnancy, particularly the risk of preterm labor due to cervical shortening. Although rescue cerclage was offered to the patient, this option was declined. She was therefore placed on bedrest for two weeks with follow-up ultrasonography for assessment of cervical length.

At 19^2/7 ^weeks gestation the patient experienced abdominal cramping and non-purulent blood-tinged vaginal discharge. The patient remained afebrile. She was hospitalized and placed on bed rest in Trendelenburg position after sterile speculum exam found the cervix two cm dilated with protruding "hourglass membranes".

One day later, amniotic membranes had fully retracted and were no longer visible above a closed cervix. Microscopic examination of vaginal fluid found occasional clue cells. External monitoring identified occasional uterine contractions; heart rates at ~150/min were measured for both twins. The patient was again counseled about the implications of preterm labor at this early stage, and the uncertainty of preventing further cervical dilation. After consideration of all therapeutic options (including cerclage), the patient elected tocolysis with a view to save her pregnancy. A 4 g loading dose of magnesium sulfate was administered intravenously, followed by a maintenance dose of 2 g/h. Oral metronidazole (500 mg) was given every 8 h, and 500 mg ampicillin was given intravenously every 6 h after a 2 gm loading dose according to hospital protocol. Additionally, oral indomethacin (50 mg) was given every 6 h for 3 days. Just as the magnesium sulfate was initiated, the patient experienced spontaneous rupture of membranes and prolapse of umbilical cord of twin A was noted several hours later. Fetal demise was confirmed approximately 1 h later, but there was no evidence of labor or infection over the next 24 h. After discussing the potential dangers of prolonged rupture of membranes, retention of dead fetus, maternal sepsis, the potential for prolonged hospitalization, need for hysterectomy and risk of death, the patient elected to continue limited oxytocin augmentation in an attempt to deliver twin A and salvage twin B. The risk of losing both fetuses was carefully discussed, and the patient agreed with this management approach despite the acknowledged uncertainty of outcome.

After 8 h of oxytocin therapy, a stillborn female fetus (319 g) was delivered. The placenta remained *in situ *and the umbilical cord of twin A was divided and ligated near the cervix with 3-0 chromic gut suture. Oxytocin was immediately discontinued. Monitoring of twin B confirmed stable heart tones and appropriate fetal movement throughout delivery of the non-viable twin. Intravenous ampicillin and metronidazole were continued postpartum but MgSO_4 _was not reinitiated. Immediately following delivery of twin A, sterile speculum exam found a closed cervix with the umbilical cord completely retracted *in utero*. Maternal vital signs remained stable and she was discharged home one week later on full bed rest, preterm labor precautions, and oral amoxicillin-clavulanate (875 mg) twice daily × 5 d.

At 25 weeks gestation, 12 mg betamethasone was administered intramuscularly with an additional dose 24 h later. There was no evidence of infection or coagulopathy at biweekly clinical evaluations, which included serial ultrasounds until 34 weeks to assess cervical length. Formal biophysical profiles (BPP) began at 28 weeks, when the estimated fetal weight was 1250 g and the BPP score was 8/8. At this time mild uterine irritability was detected and 5 mg oral terbutaline was given every 4 h until 34 weeks. Uterine activity was reduced following oral terbutaline therapy.

**Figure 1:**
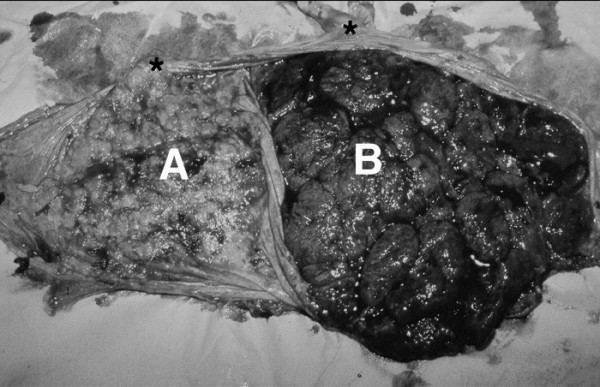
Gross post-delivery image of dichorionic-diamnionic placenta showing sites of umbilical cord insertions (*). The placenta associated with the nonviable 19-week spontaneous abortion (A) demonstrates atrophy, in contrast with normal placenta from the aftercoming liveborn twin (B).

Spontaneous labor began at 35 1/7 weeks. At readmission, the cervix was 5 cm dilated with intact membranes and vertex presentation. Epidural anesthesia was established, an amniotomy was performed, and the patient had a normal progress of labor. She delivered a viable male infant weighting 2894 g (1 and 5 min Apgar 9 and 9, respectively) over an intact perineum. Approximately 5 min later, two placentas were delivered spontaneously (Figure 1). The postpartum course was uncomplicated; mother and baby were discharged home in stable condition 48 h later.

## Conclusion

The incidence of twins has increased substantially due to advancements in the assisted reproductive technologies [1]. With more twin gestations have come more variations on the twin birth experience, sometimes including very prolonged intervals between deliveries of the twins themselves. Important risks associated with asynchronous twins include ascending infection and subsequent chorioamnionitis after delivery of the first twin. For our patient, the development of intrauterine infection and possible septic abortion of twin B was carefully discussed [2], as was the potential for severe coagulopathy in the setting of undelivered placental tissue [3].

Controversy persists on the matter of how best to manage such patients with delayed delivery interval of the second twin, perhaps because of the overall rarity of this clinical presentation. Since cases with positive outcomes (including the current report) are more likely to appear in the medical literature than those describing an unsatisfactory result, a publishing bias may favor the former.

While the longest known intertwin delivery interval with cerclage is 153 d [4], our experience of twin pregnancy with a 110 d delivery interval illustrates the potential for a satisfactory obstetrical course without cerclage. Even as our report describes one of the longest known inter-twin delivery intervals managed without cerclage, others have described even longer delivery intervals, also without cerclage [5]. Our management validates the principle that when the first twin is delivered very prematurely, extending the gestational age for an undelivered co-twin is advantageous for the second twin without significant morbidity in the mother. In selected multiple gestations achieved with medical assistance (*i.e*., conceptions following use of the advanced reproductive technologies), some authors have suggested that attempts to prolong the pregnancy following spontaneous abortion or extremely premature birth of one fetus is efficacious and justified [6].

## Competing interests

The author(s) declare that they have no competing interests.

## Authors' contributions

LMG and SAW were the resident physicians associated with the case. SAS was the lead obstetrician and primary physician at delivery. ESS conceived of the research, directed the residents, and coordinated manuscript revisions.

## Pre-publication history

The pre-publication history for this paper can be accessed here:


